# A Sticky Situation: Variable Agreement Between Platelet Function Tests Used to Assess Anti-platelet Therapy Response

**DOI:** 10.3389/fcvm.2022.899594

**Published:** 2022-07-01

**Authors:** Hirotomo Nakahara, Tania Sarker, Christina L. Dean, Susana L. Skukalek, Roman M. Sniecinski, C. Michael Cawley, Jeannette Guarner, Alexander Duncan, Cheryl L. Maier

**Affiliations:** ^1^Department of Pathology and Laboratory Medicine, Emory University School of Medicine, Atlanta, GA, United States; ^2^Department of Neurosurgery, Emory University School of Medicine, Atlanta, GA, United States; ^3^Department of Anesthesiology, Emory University School of Medicine, Atlanta, GA, United States

**Keywords:** antiplatelet therapy, aspirin, P2Y_12_ inhibitor, platelet function test, aggregometry, impedance, VerifyNow, AspirinWorks

## Abstract

**Background:**

Platelet function testing to monitor antiplatelet therapy is important for reducing thromboembolic complications, yet variability across testing methods remains challenging. Here we evaluated the agreement of four different testing platforms used to monitor antiplatelet effects of aspirin (ASA) or P2Y_12_ inhibitors (P2Y12-I).

**Methods:**

Blood and urine specimens from 20 patients receiving dual antiplatelet therapy were analyzed by light transmission aggregometry (LTA), whole blood aggregometry (WBA), VerifyNow PRUTest and AspirinWorks. Result interpretation based on pre-defined cutoff values was used to calculate raw agreement indices, and Pearson's correlation coefficient determined using individual units of measure.

**Results:**

Agreement between LTA and WBA for P2Y12-I-response was 60% (*r* = 0.65, high-dose ADP; *r* = 0.75, low-dose ADP). VerifyNow agreed with LTA in 75% (*r* = 0.86, high-dose ADP; *r* = 0.75, low-dose ADP) and WBA in 55% (*r* = 0.57) of cases. Agreement between LTA and WBA for ASA-response was 45% (*r* = 0.09, high-dose collagen WBA; *r* = 0.19, low-dose collagen WBA). AspirinWorks agreed with LTA in 60% (*r* = 0.32) and WBA in 35% (*r* = 0.02, high-dose collagen WBA; *r* = 0.08, low-dose collagen WBA) of cases.

**Conclusions:**

Overall agreement varied from 35 to 75%. LTA and VerifyNow demonstrated the highest agreement for P2Y12-I-response, followed by moderate agreement between LTA and WBA. LTA and AspirinWorks showed moderate agreement for aspirin response, while WBA showed the weakest agreement with both LTA and AspirinWorks. The results from this study support the continued use of LTA for monitoring dual antiplatelet therapy, with VerifyNow as an appropriate alternative for P2Y12-I-response. Integration of results obtained from these varied testing platforms with patient outcomes remains paramount for future studies.

## Introduction

Platelets are essential mediators of hemostasis, playing a fundamental role in clot formation under both physiologic and pathophysiologic processes. Platelet aggregation has long been recognized as a major driver of ischemic arterial events, including in coronary artery disease, peripheral arterial disease, and cerebrovascular events, as well as in complications associated with interventions such as angioplasty and stenting. For this reason, antiplatelet therapy, with either a single agent or combined dual antiplatelet therapy (DAPT), remains a key strategy for preventing thrombotic complications in patients with vascular disease (1–3). A standard DAPT regimen of aspirin (ASA) in combination with a P2Y_12_ inhibitor (P2Y12-I), like clopidogrel, has been used widely in both cardiac and neurologic populations undergoing stent placement.

The association of high platelet reactivity with increased risk of major adverse cardiovascular events has driven interest in utilizing platelet function testing (PFT) to guide antiplatelet therapy. Although major clinical trials in the post-percutaneous coronary intervention (PCI) cardiac patient population have failed to consistently demonstrate improved clinical outcomes in escalating DAPT based on PFT results (4, 5), current recommendations by the American College of Cardiology/American Heart Association (ACC/AHA) suggest PFT may provide useful prognostic data for cardiovascular risk prediction after elective PCI in stable CAD (6, 7). There is also interest in using PFT to monitor DAPT for high-risk patients in other clinical settings, such as neurovascular stenting (8, 9). Multiple testing platforms are available to measure platelet function and antiplatelet drug efficacy, but the lack of standardization necessitates that individual laboratories choose among the different platforms and methodologies, as well as their interpretation for clinical application (10, 11).

Platelet aggregometry by either light transmittance or electrical impedance is regarded as the optimal methodology for measuring platelet function. Light Transmission Aggregometry (LTA) tests platelet aggregation responses using optical density measurement following stimulation by various external agonists, such as epinephrine, arachidonic acid (AA), adenosine phosphate (ADP), or collagen (3). LTA remains the historic “gold standard” testing platform for measuring platelet function, despite some limitations (12). Specifically, LTA is expensive, requires specialized technical training, and relies on multi-step protocols that include centrifugation to produce platelet-rich plasma (13). In addition, LTA may exclude giant, hypo or hyperactive platelets from evaluation (14). Whole Blood Aggregometry (WBA) by electrical impedance measures the increase in electrical resistance generated by the aggregation of platelets between two electrodes after addition of various agonists. In comparison to LTA, WBA requires minimal sample processing, avoiding the centrifugation process and thus reducing the potential for erroneous platelet activation or loss of contribution from specific platelet populations like giant platelets (15). Furthermore, WBA assesses platelets in their native environment alongside red blood cells and leukocytes, thereby preserving red cell function to modulate ADP metabolism and platelet response. Thus, WBA may be considered more sensitive in its ability to detect inhibition of ADP-induced platelet aggregation by antiplatelet agents such as P2Y_12_ inhibitors (P2Y12-I) (14, 15).

Tests such as VerifyNow (Werfen) and AspirinWorks (Corgenix) are increasingly popular due to ease of administration, fast turnaround time, and lack of sample processing. The VerifyNow assay detects agglutination of fibrinogen-coated beads in response to agonist by an increase in light transmission. It is commonly used for detecting antiplatelet drug resistance to P2Y12-I via high-dose ADP, or resistance to ASA-inhibition via the AA pathway. At our institution, we only offer the high-dose ADP cartridge for VerifyNow to assess P2Y12-I and do not use the alternate cartridge necessary for testing ASA response. The VerifyNow measures and reports P2Y12-I response as P2Y12 Reaction Units (PRU). AspirinWorks is an ELISA-based assay performed on urine specimens that detects the chemical biomarker 11-dehydrothromboxane B2, a downstream product of AA metabolism by activated platelets. Thus, low levels of 11-dehydrothromboxane B2 suggest ASA is effectively reducing thromboxane production and resulting in efficacious antiplatelet response.

At our institution, we perform full platelet aggregation profiles via LTA for hematology patients in whom a platelet defect is suspected. To support the clinical needs of our interventional radiology teams, we devised a truncated LTA-based test with a limited set of agonists (high-dose ADP, low-dose ADP, and AA), called the Platelet Inhibitor of Platelet Aggregation (PIPA), to assess patients' DAPT response. The primary users of this panel are interventional neuroradiologists with patients undergoing placement of flow-diversion devices for treatment of intracranial aneurysms, though it is used for cardiac and hematologic patients as well. To improve our DAPT response test algorithm, we devised this study to compare the agreement among the LTA-based PIPA with WBA, VerifyNow PRU Test, and AspirinWorks, for potential implementation into our laboratory workflow.

## Method

Blood and urine samples from 20 patients receiving DAPT prior to neurovascular stenting were collected prospectively. Patient demographic and clinical information is provided in the Supplementary Table 1. Blood samples were analyzed by LTA using platelet-rich plasma on the Helena AggRAM and by WBA measuring electrical impedance using the Chrono-log Lumi-aggregometer to assess ASA and P2Y12-I response. Agonists were chosen and responses interpreted based on guidelines established by internal laboratory validation (LTA) or by following manufacturer-provided thresholds (WBA). VerifyNow PRU Test (Werfen, Barcelona, Spain) was performed on whole blood to assess P2Y12-I effect, and AspirinWorks (Corgenix, Broomfield, CO, USA) was performed on urine specimens to assess ASA effect, each following manufacturers' guidelines. Result interpretation for all testing platforms is summarized in the Supplementary Table 2.

For LTA, the maximum amplitude (MA) of platelet aggregation was determined using high-dose (20 μM) and low-dose (5 μM) ADP (Helena) agonist for measuring P2Y12-I response, and with AA (500 μM, Helena) for ASA response. Inhibited platelet aggregation by P2Y12-I was defined as MA ≤60% for high-dose ADP and MA ≤40% for low-dose ADP. Inhibited platelet aggregation by ASA was defined as MA ≤20%; MA >20% but ≤26% was interpreted as near optimal inhibition. For all agonists, MA values above these cutoffs were considered suboptimal platelet suppression and interpreted as uninhibited. These thresholds were previously established through an internal validation within our institution's Special Coagulation Laboratory.

For WBA, resistance (ohms) was measured with ADP (5 μM, Chronolog) for P2Y12-I response and with high-dose (5 μg/ml) and low-dose (1 μg/ml) collagen (Chronolog) for ASA response. Interpretation was based on the manufacturer's recommendations. Specifically, inhibited platelet aggregation by P2Y12-I was defined as a response of ≤5 ohms with ADP. Inhibited platelet aggregation by ASA was defined as ≤8 ohms for low-dose collagen response and ≥50% decrease between high-dose and low-dose collagen response. All other results were considered uninhibited platelet aggregation.

For the VerifyNow PRUTest, results <180 PRU (P2Y12 Reaction Units) were considered evidence of therapeutic P2Y12 inhibitor effect (inhibited), while ≥180 PRU indicated a lack of therapeutic P2Y12-I effect (uninhibited), in keeping with the manufacturer's recommendations. For AspirinWorks, detection of <1,000 pg/mg of 11-dehydrothromboxane B2 was considered evidence of therapeutic ASA effect (inhibited), 1,000–1,400 pg/mg as equivocal, and >1,400 pg/mg as lack of therapeutic ASA effect (uninhibited), per manufacturer's suggested cutoffs.

Raw agreement indices between testing platforms were calculated as the proportion of overall agreement (P_o_) between the two testing platforms being compared, where the sum of positive (inhibited/inhibited) and negative (uninhibited/uninhibited) agreement were divided by the total number of patients (*n* = 20). For the ASA response test comparisons, results of “near optimal inhibition” were interpreted as “inhibited,” and “equivocal” treated as “uninhibited” for data analysis. This was based on the clinical practice of our treating-providers, where medication dosage is adjusted (i.e., escalated) for patients with “equivocal” results but not for “near optimal” results. Contingency table analysis was done by Fisher's exact test. Pearson's correlation coefficient was determined using actual units of measurement for each testing platform. All calculations and statistical analysis were performed using Microsoft Excel and GraphPad Prism software.

## Results

### Comparison of P2Y12-I Response Tests

Summary results of P2Y12-I response in 20 patients assessed by LTA, WBA, and VerifyNow are shown in Table 1. There was a notable lack of agreement across the three different testing platforms. Optimal P2Y12-I response was detected in 8/20 patients by LTA, 6/20 patients by WBA, and 13/20 patients by VerifyNow. Suboptimal P2Y12-I response was detected in 12/20 patients by LTA, 14/20 patients by WBA, and 7/20 patients by VerifyNow.

**Table 1:** Summary of P2Y12-I-response results compared across three testing platforms.

**Patient (*n* = 20)**	**LTA (MA %)**			**WBA (ohms)**		**VerifyNow (PRU)**	
	**ADP (20 μM)**	**ADP (5 μM)**	**Interp**	**ADP (5 μM)**	**Interp**	**PRU test**	**Interp**
A1	50	25	Inhibited	7	Uninhibited	89	Inhibited
A2	84	69	Uninhibited	17	Uninhibited	211	Uninhibited
A3	28	18	Inhibited	6	Uninhibited	2	Inhibited
A4	33	26	Inhibited	2	Inhibited	4	Inhibited
A5	77	59	Uninhibited	12	Uninhibited	162	Inhibited
A6	65	51	Uninhibited	12	Uninhibited	56	Inhibited
A7	40	36	Inhibited	6	Uninhibited	59	Inhibited
A8	65	60	Uninhibited	11	Uninhibited	143	Inhibited
A9	31	24	Inhibited	2	Inhibited	2	Inhibited
A10	60	42	Uninhibited	4	Inhibited	159	Inhibited
A11	75	56	Uninhibited	9	Uninhibited	202	Uninhibited
A12	36	30	Inhibited	9	Uninhibited	47	Inhibited
A13	74	58	Uninhibited	17	Uninhibited	293	Uninhibited
A14	64	42	Uninhibited	10	Uninhibited	260	Uninhibited
A15	62	47	Uninhibited	4	Inhibited	167	Inhibited
A16	74	59	Uninhibited	8	Uninhibited	207	Uninhibited
A17	55	37	Inhibited	2	Inhibited	43	Inhibited
A18	86	77	Uninhibited	18	Uninhibited	265	Uninhibited
A19	57	29	Inhibited	6	Uninhibited	110	Inhibited
A20	67	42	Uninhibited	1	Inhibited	228	Uninhibited
		**# Positive agreement**		**# Negative agreement**		**Proportion of overall**	
		**(Both tests inhibited)**		**(Both tests uninhibited)**		**agreement (P_O_)**	
LTA vs. WBA		3		9		60%	
LTA vs. VerifyNow (PRU)		8		7		75%	
WBA vs. VerifyNow (PRU)		5		6		55%	

*ADP, adenosine diphosphate; LTA, Light transmission aggregometry; PRU, P2Y12 Reaction Units; WBA, Whole blood aggregometry*.

The proportion of overall agreement (P_o_) ranged between 55 and 75% among the three P2Y12-I response tests compared. P_o_ between LTA and WBA was 60%, P_o_ between LTA and VerifyNow PRU Test was 75%, and P_o_ between WBA and VerifyNow PRU Test was 55%. Contingency table analysis by Fisher's exact test showed statistical significance for LTA vs. VerifyNow (*p* = 0.0147). The other two comparisons failed to achieve statistical significance (LTA vs. WBA *p* = 0.6424, WBA vs. VerifyNow PRU *p* = 0.3544), most likely due to the limited number of patients tested in this study.

**Figure 1:**
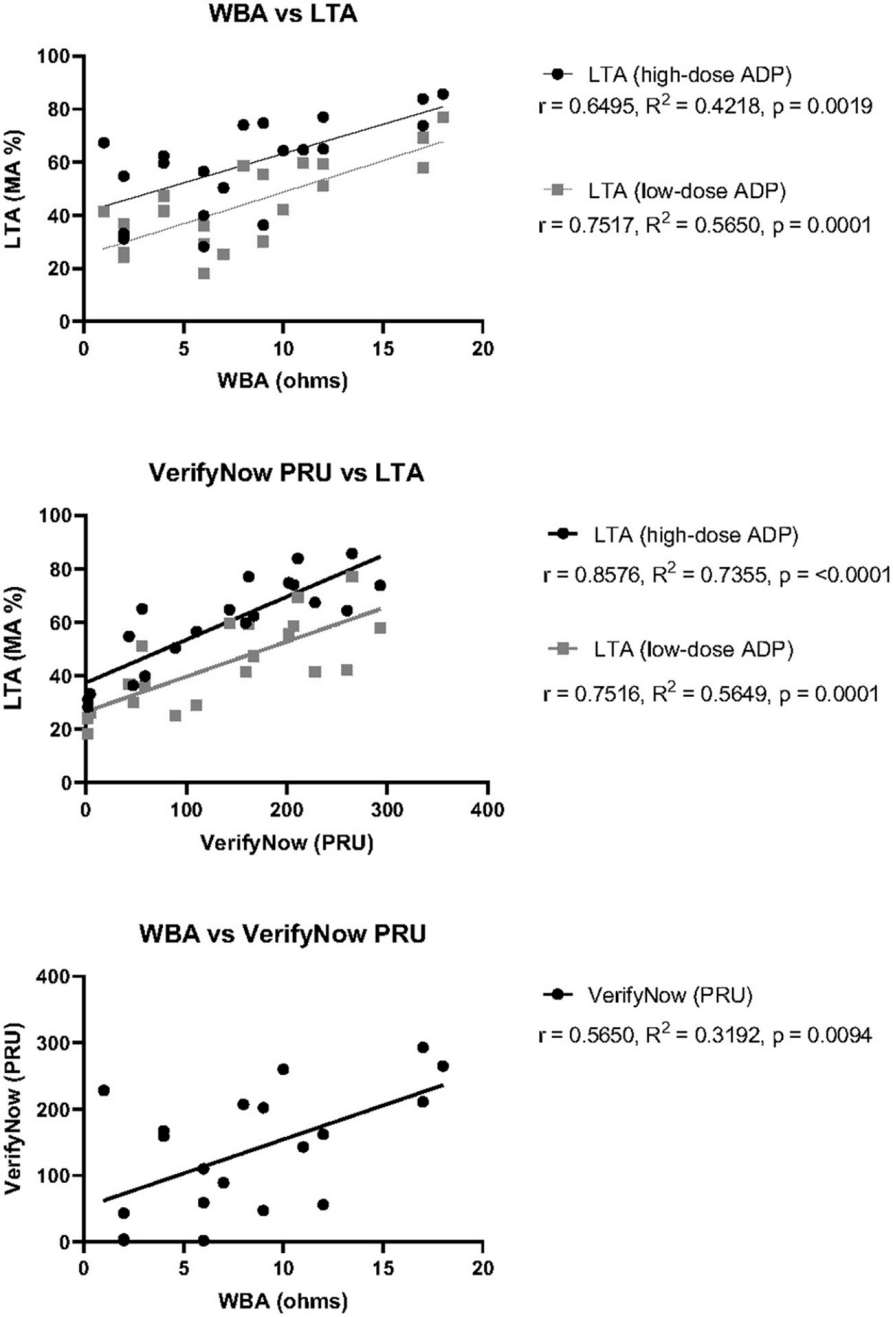
Correlation of P2Y12-I response test measurements between three test platforms. Pearson's correlation coefficient (r), goodness of fit by simple linear regression (R^2^), and statistical significance (p) was calculated for each pair of tests using the raw measurement values obtained for each patient. Top: WBA against either LTA with high-dose ADP (black) or low- dose ADP (gray). Middle: VerifyNow PRU Test against either LTA with high-dose ADP (black) or low-dose ADP (gray). Bottom: WBA against VerifyNow PRU Test.

To investigate whether the variability in overall agreement could be attributable to differences in the pre-defined cutoff values established for interpretation, the correlation between exact measurement units was also calculated (Figure 1). There was moderate, positive correlation between WBA and LTA with high-dose ADP, where *r* = 0.65, *R*^2^ = 0.42, and *p* = 0.0019. The positive correlation was stronger between WBA and LTA with low-dose ADP, showing *r* = 0.75, *R*^2^ = 0.57, and *p* = 0.0001. Comparison between VerifyNow and LTA with high-dose ADP demonstrated the strongest positive correlation among all tests for P2Y12-I response, with *r* = 0.86, *R*^2^ = 0.74, and *p* ≤ 0.0001. VerifyNow and LTA with low-dose ADP showed moderately positive correlation of *r* = 0.75, *R*^2^ = 0.57, and *p* = 0.0001, which was nearly identical to the correlation between WBA and LTA with low-dose ADP. Finally, comparison between WBA and VerifyNow showed the lowest positive correlation among all P2Y12-I tests, with *r* = 0.5650, *R*^2^ = 0.3192, and *p* = 0.0094. Overall, all three test platforms for P2Y12-I response demonstrated varying degrees of statistically significant, positive correlation among themselves, despite the variability in overall agreement.

### Comparison of ASA Response Tests

Summary results of ASA response in 20 patients compared across LTA, WBA, and AspirinWorks are shown in Table 2. For data analysis, the LTA result of “near optimal” for 3 patients (A3, A10, and A16) was considered equivalent to “optimal” platelet suppression, and the AspirinWorks result of “equivocal" for 6 patients (A3, A5, A7, A10, A12, and A14) was considered equivalent to “suboptimal.” These considerations were defined ahead of data collection and analysis, and align with the laboratory's interpretation used for clinical decision-making. There was notable lack of agreement across the three different testing platforms for ASA response, similarly to comparison among the P2Y12-I response methods. Optimal ASA response was detected in 12/20 patients by LTA, 11/20 patients by WBA, and 8/20 by AspirinWorks. Suboptimal ASA response was detected in 8/20 patients by LTA, 9/20 patients by WBA, and 12/20 patients by AspirinWorks.

**Table 2:** Summary of ASA-response results compared across three testing platforms.

**Patient (*n* = 20)**	**LTA (MA %)**		**WB (ohms)**			**AspirinWorks (pg/mg)**	
	**AA (500 μM)**	**Interp**	**Collagen (1 μg/ml)**	**Collagen (5 μg/ml)**	**Interp**	**AspirinWorks test**	**Interp**
A1	7	Inhibited	10	27	Uninhibited	1065	Inhibited
A2	32	Uninhibited	5	11	Inhibited	1599	Uninhibited
A3	22	Inhibited	6	16	Inhibited	1266	Equivocal
A4	64	Uninhibited	18	19	Uninhibited	2833	Uninhibited
A5	32	Uninhibited	6	12	Inhibited	1189	Equivocal
A6	35	Uninhibited	8	17	Inhibited	711	Inhibited
A7	29	Uninhibited	10	20	Uninhibited	1284	Equivocal
A8	30	Uninhibited	13	22	Uninhibited	545	Inhibited
A9	14	Inhibited	9	14	Uninhibited	640	Inhibited
A10	24	Inhibited	6	11	Uninhibited	1030	Equivocal
A11	67	Uninhibited	6	21	Inhibited	1467	Uninhibited
A12	17	Inhibited	6	19	Inhibited	1327	Equivocal
A13	14	Inhibited	6	14	Inhibited	1852	Uninhibited
A14	19	Inhibited	7	13	Uninhibited	1187	Equivocal
A15	28	Uninhibited	5	12	Inhibited	1838	Uninhibited
A16	23	Inhibited	8	7	Uninhibited	654	Inhibited
A17	22	Inhibited	6	14	Inhibited	3427	Uninhibited
A18	15	Inhibited	18	22	Uninhibited	960	Inhibited
A19	20	Inhibited	3	15	Inhibited	912	Inhibited
A20	26	Inhibited	2	6	Inhibited	806	Inhibited
		**# Positive agreement**		**# Negative agreement**		**Proportion of overall**	
		**(Both tests Inhibited)**		**(Both tests Uninhibited or Equivocal)**		**agreement (P_O_)**	
LTA vs. WBA		6		3		45%	
LTA vs. AspirinWorks		6		6		60%	
WBA vs. AspirinWorks		3		4		35%	

*AA, arachidonic acid; LTA, Light transmission aggregometry; WBA, Whole blood aggregometry*.

Proportion of overall agreement (P_o_) ranged between 35 and 60% among the three tests of ASA response. P_o_ between LTA and WBA was 45%, P_o_ between LTA and AspirinWorks was 60%, and P_o_ between WBA and AspirinWorks was 35%. Contingency table analysis by Fisher's exact test failed to achieve the statistical significance threshold of *p* < 0.05 in any of the three comparisons (LTA vs. WBA *p* = 0.6699, LTA vs. AspirinWorks *p* = 0.3729, WBA vs. AspirinWorks *p* = 0.3618).

**Figure 2:**
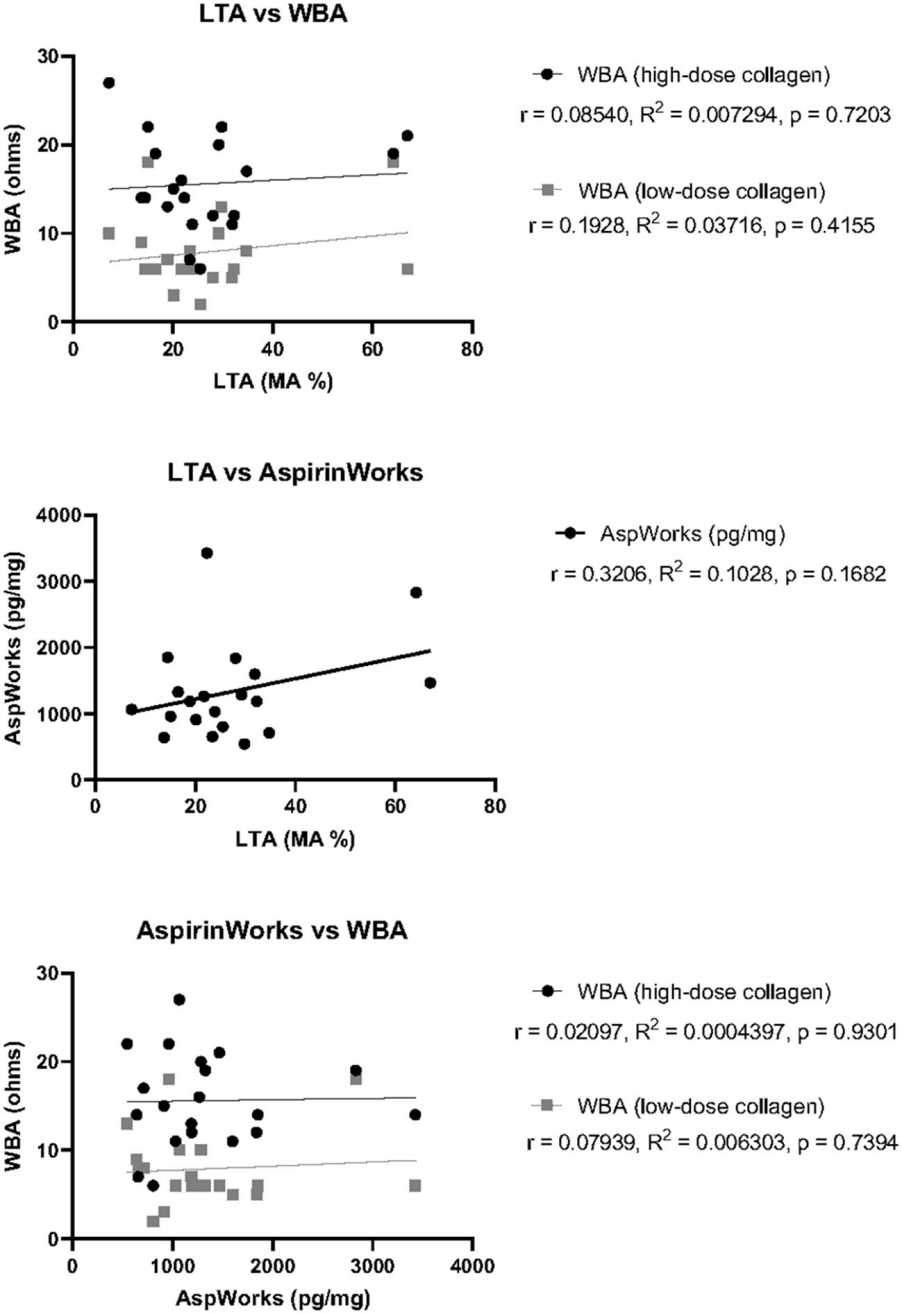
Correlation of ASA response test measurements between three test platforms. Pearson's correlation coefficient (r), goodness of fit by simple linear regression (R^2^), and statistical significance (p) was calculated for each pair of tests using the raw measurement values obtained for each patient. Top: LTA against either WBA with high-dose collagen (black) or low- dose collagen (gray). Middle: LTA against AspirinWorks. Bottom: AspirinWorks against either WBA with high-dose collagen (black) or low-dose collagen (gray).

Similarly to analysis of P2Y12-I response testing, we investigated whether the variability in overall agreement for ASA responsiveness could be attributable to difference in the pre-defined cutoff values established for interpretation by calculating the correlation between measured units for each testing platform (Figure 2). There was no correlation between LTA and high-dose collagen WBA (*r* = 0.08540, *R*^2^ = 0.007294, and *p* = 0.7203) or LTA and low-dose collagen WBA (*r* = 0.1928, *R*^2^ = 0.03716, and *p* = 0.4155). A lack of correlation was also determined between AspirinWorks and high-dose collagen WBA (*r* = 0.02097, *R*^2^ = 0.0004397, and *p* = 0.9301) as well as AspirinWorks and low-dose collagen WBA (*r* = 0.07939, *R*^2^ = 0.006303, and *p* = 0.7394). Finally, there was perhaps hint of a weak, positive correlation between LTA and AspirinWorks, although it failed to reach statistical significance (*r* = 0.3206, *R*^2^ = 0.1028, and *p* = 0.1682). The lack of overall agreement among the ASA response test platforms was consistent with the lack of statistically significant correlation.

## Discussion

Overall agreement of the platelet function testing platforms used for monitoring DAPT in this study ranged between 50 and 75% for P2Y12-I response testing by LTA, WBA, and VerifyNow, and 35–60% for ASA response testing by LTA, WBA, and AspirinWorks. There was moderate to strong positive correlation in measurement units among the P2Y12-I response tests (*r* = 0.57–0.86), but a lack of correlation observed in measurement units among the ASA response tests (*r* = 0.02–0.32, but all with p > 0.05). The variability in agreement and correlation is similar to other studies comparing multiple PFT platforms for ASA and P2Y12-I response (16–20), and likely attributable to the differences in methodology underlying each testing platform and the lack of standardized laboratory definitions for adequate platelet suppression in response to DAPT (11, 21).

For P2Y12-I response testing, WBA offers theoretical advantages over LTA by better representing *in vivo* platelet function and increased sensitivity to P2Y12-I effect (14, 15). In our study only 6/20 patients were considered to have optimal platelet suppression by WBA, compared to 8/20 patients by LTA, with a moderate level of overall agreement (50% for P2Y12-I). Given the strong correlation in measurement units for P2Y12-I response (*r* = 0.65–0.75), there is potential for improved agreement between LTA and WBA with modification of cut-off values used for interpretation, particularly those driven by integration with clinical outcomes.

The VerifyNow PRU Test has been widely adopted due to its ease of use as a waived, point-of-care test, and the ability to compare results across laboratories and health-systems (3, 21). Specifically, VerifyNow testing has enabled establishment of cut-off values for drug efficacy, which have been supported by multiple clinical trials of PFT-guided antiplatelet therapy in cardiac patients post-PCI (7). Our comparison of VerifyNow with LTA demonstrated the strongest agreement and correlation (75%, and *r* = 0.75–0.86) for P2Y12-I response among all tests we assessed, likely a reflection of their similar methodologies (22, 23). The consistent, positive correlation between LTA and VerifyNow has been borne out in multiple studies despite some variability in agreement (18–20).

For assessment of antiplatelet ASA response, AspirinWorks demonstrated superior level of agreement with LTA (60%) compared to agreement between WBA and LTA (45%), but with no statistically significant correlation in measurement units across platforms. Other comparison studies have also noted a lack of agreement and correlation among tests of ASA responsiveness. In the case of AspirinWorks, urinary 11-dehydrothromboxane B2 measurements may be impacted by potential contribution of thromboxane A2 synthesis from non-platelet sources (16, 17, 24). Nevertheless, AspirinWorks offers the benefit of being an FDA-cleared test allowing for at-home urine collection by patients. Additional studies investigating clinical outcomes associated with ASA responses measured by LTA vs. AspirinWorks or WBA are certainly warranted.

It should be noted that the combined agreement between WBA with VerifyNow PRU Test (55%) or with AspirinWorks (35%) was much lower than that of LTA with VerifyNow PRU Test (75%) or with AspirinWorks (60%). For that reason, we have maintained LTA for testing DAPT response in patients at our institution and have not yet implemented a WBA-based PIPA to assess P2Y12-I and ASA response, despite the potential workflow benefit associated with using whole blood-based assays.

We acknowledge several limitations of our study, including that all samples were from patients undergoing neurologic stenting at a single institution and without clinical outcome validation. In addition, it is worth noting that none of the tests confirmed DAPT effect in all 20 patients, for whom DAPT was prescribed. This may be due to any number of factors, including true medication non-responsiveness, drug-drug interactions, preanalytical testing variables (e.g., activated platelets in specimen before agonist addition), or medication non-compliance. How these factors variably impact the performance of each assay was unable to be determined in our study. Notably, ACC/AHA guidelines state a preference for point of care devices for PFT, but the relevance to other patient populations remains uncertain. Our findings are generally applicable to the laboratory variability of PFT in assessing DAPT response and highlight the “sticky situation” encountered by laboratories and health-systems when choosing among the diverse PFT options available.

In conclusion, our study supports the continued use of “gold standard” LTA for monitoring platelet function in patients on DAPT. VerifyNow PRU Test offers an appropriate alternative for testing P2Y12-I effect, with the possibility of testing ASA suppression via the VerifyNow Aspirin Test; however, the agreement and correlation between the latter with LTA was unable to be assessed. We continue to use AspirinWorks as an adjunct test for ASA effect in the appropriate clinical setting. In our hands, the relatively inferior performance of WBA outweighed the potential advantage of implementing WBA-based testing in our laboratory without further assay optimization. Additional studies integrating clinical outcomes with results generated from these various testing platforms are needed to shed light on the appropriateness and optimization of DAPT testing.

## Data Availability Statement

The original contributions presented in the study are included in the article/Supplementary Material, further inquiries can be directed to the corresponding author.

## Ethics Statement

Ethical review and approval was not required for the study on human participants in accordance with the local legislation and institutional requirements. Written informed consent for participation was not required for this study in accordance with the national legislation and the institutional requirements.

## Author Contributions

SS, JG, AD, and CM: conception and design. HN, TS, CD, SS, and CM: data acquisition and analysis. HN, TS, SS, RS, JG, AD, and CM: data interpretation and drafting of the manuscript. HN, RS, CC, JG, AD, and CM: revision of the manuscript. All authors contributed to the article and approved the submitted version.

## Funding

This work was supported by institutional funds as part of a quality project. CM is supported by NIH/NHLBI K99 HL150626. CD and TS received support from NIH training grant T32HL069769.

## Conflict of Interest

The authors declare that the research was conducted in the absence of any commercial or financial relationships that could be construed as a potential conflict of interest.

## Publisher's Note

All claims expressed in this article are solely those of the authors and do not necessarily represent those of their affiliated organizations, or those of the publisher, the editors and the reviewers. Any product that may be evaluated in this article, or claim that may be made by its manufacturer, is not guaranteed or endorsed by the publisher.
