# Supplementary material for: A Sticky Situation: Variable Agreement Between Platelet Function Tests Used to Assess Anti-platelet Therapy Response
Source: Front Cardiovasc Med. 2022 Jul 1;9:899594. doi: 10.3389/fcvm.2022.899594 (PMC9283921; doi:10.3389/fcvm.2022.899594)
Supplement: Supplementary file 2 [file Table_2.docx]

**Supplemental Table 2. Overview of Testing Platform Interpretations***

| Antiplatelet Agent | Testing Platform | Result Thresholds | Therapeutic Response | Interpretation |
| --- | --- | --- | --- | --- |
| P2Y12 Inhibitor | LTA | ADP (20µM) MA<60% and  ADP (5µM) MA<40% | Optimal | Inhibited |
|  |  | ADP (20µM) MA > 60% or  ADP (5µM) MA > 40% | Suboptimal | Uninhibited |
|  | WBA | ADP (5µM) < 5 ohms | Optimal | Inhibited |
|  |  | ADP (5µM) > 5 ohms | Suboptimal | Uninhibited |
|  | VerifyNow  PRU Test | <180 PRU | Optimal | Inhibited |
|  |  | >180 PRU | Suboptimal | Uninhibited |
| Aspirin | LTA | AA (500µM) MA < 20% | Optimal | Inhibited |
|  |  | AA (500µM) MA < 26% | Near Optimal | Inhibited |
|  |  | AA (500µM) MA > 27% | Suboptimal | Uninhibited |
|  | WBA | >50% reduction between 1ug/mL versus 5ug/mL collagen | Optimal | Inhibited |
|  |  | <50% reduction between 1ug/mL versus 5ug/mL collagen or >8ohms to 1ug/mL collagen | Suboptimal | Uninhibited |
|  | AspirinWorks | <1000 pg/mg 11-dehydrothromboxane B2 | Optimal | Inhibited |
|  |  | 1000-1400 pg/mg 11-dehydrothromboxane B2 | Equivocal | Equivocal |
|  |  | >1400 pg/mg 11-dehydrothromboxane B2 | Suboptimal | Uninhibited |

*Determination of therapeutic responses and final interpretation (inhibited versus uninhibited) were based off of result thresholds established by either internal validation (LTA) or manufacturer’s guidelines (WBA, VerifyNow PRU Test, AspirinWorks). AA = arachidonic acid, ADP = adenosine diphosphate, LTA = Light transmission aggregometry, PRU = P2Y12 Reaction Units, WBA = Whole blood aggregometry.
